# Rapid Authentication of 100% Italian Durum Wheat Pasta by FT-NIR Spectroscopy Combined with Chemometric Tools

**DOI:** 10.3390/foods9111551

**Published:** 2020-10-27

**Authors:** Annalisa De Girolamo, Salvatore Cervellieri, Erminia Mancini, Michelangelo Pascale, Antonio Francesco Logrieco, Vincenzo Lippolis

**Affiliations:** Institute of Sciences of Food Production (ISPA), CNR-National Research Council of Italy, Via G. Amendola 122/O, 70126 Bari, Italy; salvatore.cervellieri@ispa.cnr.it (S.C.); erminia.mancini@ispa.cnr.it (E.M.); michelangelo.pascale@ispa.cnr.it (M.P.); antonio.logrieco@ispa.cnr.it (A.F.L.); vincenzo.lippolis@ispa.cnr.it (V.L.)

**Keywords:** FT-NIR spectroscopy, durum wheat pasta, Italian durum wheat, authentication, geographical origin, PC-LDA, SVM, PLS-DA

## Abstract

Italy is the country with the largest durum wheat pasta production and consumption. The mandatory labelling for pasta indicating the country of origin of wheat has made consumers more aware about the consumed pasta products and is influencing their choice towards 100% Italian wheat pasta. This aspect highlights the need to promote the use of domestic wheat as well as to develop rapid methodologies for the authentication of pasta. A rapid, inexpensive, and easy-to-use method based on infrared spectroscopy was developed and validated for authenticating pasta made with 100% Italian durum wheat. The study was conducted on pasta marketed in Italy and made with durum wheat cultivated in Italy (*n* = 176 samples) and on pasta made with mixtures of wheat cultivated in Italy and/or abroad (*n* = 185 samples). Pasta samples were analyzed by Fourier transform-near infrared (FT-NIR) spectroscopy coupled with supervised classification models. The good performance results of the validation set (sensitivity of 95%, specificity and accuracy of 94%) obtained using principal component-linear discriminant analysis (PC-LDA) clearly demonstrated the high prediction capability of this method and its suitability for authenticating 100% Italian durum wheat pasta. This output is of great interest for both producers of Italian pasta pointing toward authentication purposes of their products and consumer associations aimed to preserve and promote the typicity of Italian products.

## 1. Introduction

Pasta is a key element of the Mediterranean diet providing complex carbohydrates, proteins, vitamins, mineral salts, and dietary fiber to consumers. Pasta produced from durum wheat (*Triticum durum* Desf.) is of superior cooking quality thanks to several properties, including rheological properties, texture, color, and taste [1]. A quarter of the worldwide pasta output is produced in Italy, which has the highest annual production of about 3.4 million tons/year, accounting for 6% of total Italian food industry production [2]. Italy is also the largest pasta consumer (25.3 kg per capita/year), meaning that pasta represents a strategic product in the Italian agro-food industry [2]. Over the years, the consumption of pasta has spread to other countries, thus constantly increasing the growth of the global pasta market, and pasta composition has been influenced by regional tastes and preferences.

Pasta is considered an indisputable symbol of “Made in Italy” products over the world, thanks to its nutritional value, high digestibility, good shelf life, availability through the market in many shapes, and low cost, representing a good attraction for the consumer. Italian pasta companies commonly use a mix of approximately 70% Italian and 30% imported durum wheat to guarantee pasta with excellent technological parameters. However, in the recent years, the trend of producing high-quality pasta with comparable technological properties using exclusively Italian-grown wheat has becoming gradually popular, taking advantage of the interest of consumers for locally sourced products and their concerns on the consumption of pasta made with imported wheat. Indeed, the declaration on the label that pasta has been made with 100% domestic wheat gives an added value to this commodity. Therefore, production of pasta made exclusively with Italian durum wheat represents an appealing option for some Italian Companies due to its higher retail price and higher financial benefits compared to other similar products made using both Italian and imported wheat.

Furthermore, consumer awareness about the origin of products is tightly related to global market expansion. To guarantee a high level of protection of consumers’ health and to allow consumers to make informed choices and safe use of food, the European Union (EU) has established mandatory food information, which also includes information regarding the country of origin or the place of provenance of its primary ingredient (i.e., the ingredient representing more than 50% of the food) if different from the origin of the food [3]. The “Made in Italy” trademark (or the Italian flag on the package), although identifying food products being totally made in Italy in terms of planning, manufacturing, and packaging [4], does not guarantee the Italian origin of the primary ingredient, thus favoring misleading information on food labels. To avoid any misleading information to the consumer on the true origin of a food product, the current EU regulation has been further implemented to clarify how the origin of the primary ingredient should be reported when it is different from the manufacturing origin of the food [5]. The origin of raw materials is one of the main selection criteria for consumers when choosing products, including pasta. Therefore, Italy has adopted a Ministerial Decree specifically for durum wheat pasta in which it is mandatory to indicate on the label the harvesting and the milling countries of durum wheat to enable consumers to make more informed choices [6]. If harvesting and milling take place in the territory of more than one country, the following indications may be used, depending on the origin: “EU countries”, “NON-EU countries”, ‘EU and NON-EU countries”. Furthermore, if at least 50% of the durum wheat is grown in a single country, such as Italy, it is possible to use the following wording “Italy and other EU and/or NON-EU countries” [6]. This Ministerial decree has been extended till the end of the year 2021, despite the current EU legislation [7].

Italy, and in general every country, has its own specific wheat varieties, suited to its own environmental conditions and agronomic practices [8,9]. The addressing of authenticity issues and the prevention of mislabeling regarding the geographical origin of durum wheat used for pasta production requires reliable and rapid analytical methods. In recent years, several analytical methods, classified as untargeted and targeted, in combination with different chemometric approaches, have been described for the traceability and authenticity of several food products [10,11,12,13,14,15,16,17,18]. Some of these methods for cereals and pasta, whose chemical complexity makes them very challenging from the analytical point of view, are mainly based on the use of liquid and gas chromatography–mass spectrometry, isotope ratio and elemental analysis, and spectroscopic techniques [10,11,12,13,14,15,16,17,18].

Among untargeted methods, infrared (IR) spectroscopic techniques are the ones more often used for fingerprinting analysis. IR spectroscopy techniques are rapid and cost-effective, involve less or no sample preparation, are environmentally friendly and easy to use, can be adopted for online or at-line process control, and have been successfully applied for the authentication of several food products [13,19,20,21,22]. Although they have been used to analyze cereals and derived products [9,23,24,25,26,27,28], only few authors reported the application of IR spectroscopy for the authentication of pasta [29,30,31,32,33].

The present work was aimed at developing and validating, for the first time, classification models based on Fourier transform-near infrared (FT-NIR) spectroscopy for authenticating durum wheat pasta samples made exclusively with wheat cultivated in Italy, distinguishing them from those manufactured with a mixture of wheat from different countries. To this end, a chemometric analysis was applied for the discrimination of more than 350 Italian pasta samples based on the geographical origin of the wheat used for their manufacturing, as indicated on the label.

## 2. Materials and Methods 

### 2.1. Pasta Samples

A set of 361 commercial dry durum wheat Italian pasta samples (500 g each) of 33 different commercial brands (i.e., Alce Nero, Amato premium, Armando, Arte Agricola, Barilla, Baronia, Buitoni, Cocco, Coop, De Cecco, Demeter, Divella, Ecor, Esselunga, Fior fiore Coop, Frediani, Garofalo, Granoro, Granoro Dedicato, La Dispensa, La Molisana, La Terra e il Cielo, Le Vie dei Mulini, Marella, Pasta Reale, Pasta Reggia, Riscossa, Saper di sapori, Selex, Terre e Gusti, Tre Mulini, Vivi Verde Coop, Voiello) and with different shapes were collected in Italy in the period 2018–2020. According to the information reported on the labels, 176 samples were manufactured using only durum wheat cultivated in Italy, named here “Pasta 100% ITA wheat”, while 185 pasta samples were produced with a mixture of wheat cultivated in Italy and/or abroad (reported as “Italy-EU”, “Italy-NON-EU”, “Italy-EU and NON-EU”or “EU and NON-EU” on the label) and named throughout the manuscript “Pasta MIX wheat”. Each sample was finely ground by a Retsch ZM 200 (Retsch, Haan, Germany) laboratory mill, obtaining grounded samples with particle size ≤500 μm, thoroughly homogenized, and stored at +5 °C till FT-NIR analysis.

### 2.2. FT-NIR Spectroscopy Analysis and Multivariate Statistical Analysis

FT-NIR spectroscopy analysis of grounded pasta samples (approximately 30 g) was carried out according to De Girolamo et al. [23] using the spectrometer Nicolet iS50 FT-IR (Thermo Fisher Scientific Inc., Madison, WI, USA). Spectra were recorded by using 32 interferometer sub-scans and a resolution of 4 cm^−1^ in the range between 10,000–4000 cm^−1^.

Before performing multivariate analysis, maximum normalization followed by standard normal variate (SNV) and mean centering were applied to FT-NIR spectral data to reduce the spectral baseline shift, improve the signal-to-noise ratio, and remove light scatter influence [34]. Multivariate statistical analyses, i.e., principal component analysis (PCA), principal component-linear discriminant analysis (PC-LDA), partial least-squares discriminant analysis (PLS-DA), and support vector machine (SVM), were conducted with The Unscrambler^®^ X, v10.1, software (CAMO Software AS, Oslo, Norway, 2011) [35]. The Mann–Whitney U Test, performed on the content (according to the label information of the collected pasta samples) of proteins, carbohydrates, lipids, fiber, and salt, was carried out using Statistica 6.0 (StatSoft, Tulsa, OK, USA).

#### 2.2.1. Principal Component Analysis (PCA)

PCA is an unsupervised pattern recognition approach that was firstly applied to raw spectra to explore data and to recognize potential clustering (similarities and differences) of the pasta samples based on the wheat origin of the two classes (i.e., “Pasta 100% ITA wheat” and “Pasta MIX wheat”). The data matrix used for the PCA consisted of 1557 columns (corresponding to the wavenumbers recorded every 4 cm^−1^ in the range from 10,000 to 4000 cm^−1^) and of 361 rows (corresponding to the number of samples). Outliers were detected by using the graphical tools of the Unscrambler^®^ X software, i.e., the Hotelling T² line plot, using a critical limit of *p*-value < 5%, and the influence plot, displaying outlier samples as those having both high leverage and high residuals variance. No outliers were detected, even though 12 samples showed high leverage, while 5 different samples showed high residuals. The correlation loading plot was also used to investigate FT-NIR variables with correlation values within +0.7 and +1 and −0.7 and −1 (values were arbitrarily defined) that were selected as those contributing to the differentiation between the two classes. Then, the entire spectra and the selected variables were pre-processed before performing a new PCA. The Kennard–Stone (KS) algorithm was applied to each FT-NIR range (entire and partial) to split the sample set in a training set (2/3 of the total) to calibrate the models and a test set (1/3 of the total) to validate the prediction ability of the suggested models [36] (Table 1).

#### 2.2.2. Principal Component-Linear Discriminant Analysis (PC-LDA)

The PC-LDA supervised approach was used to classify samples based on the origin of the wheat according to the label information. The final PC-LDA model was carried out by applying PCA and choosing the first PCs maximizing the performance of the model. Considering that the number of variables should not exceed (*n* − *g*)/*3*, where *n* is the number of objects in the training set (i.e., 238 samples), and *g* is the number of categories (2 classes) [37], the maximum number of PCs should be 78. The numbers of PCs maximizing the performance of the model was 20. The comparison of the Mahalanobis distance of each spectrum from the two classes of the model assigned the belonging class. Specifically, samples were classified into class “Pasta 100% ITA wheat” or “Pasta MIX wheat” considering the lowest distance between the origin of the discrimination plot and the projection of the sample.

#### 2.2.3. Partial Least-Squares Discriminant Analysis (PLS-DA)

The supervised PLS-DA tool is a method that compresses the spectral data into orthogonal structures called latent variables (LVs), which describe the maximum covariance between the spectral information and the reference values. The chosen number of LVs was the one providing the lowest prediction error in cross-validation (20 segments equivalent to leave-11-out). A total of 9 LVs, explaining 56% of the total variance, guaranteed the optimal model complexity. The PLS-DA model is based on the PLS algorithm, where the dependent variable *y* is categorical and represents samples’ class membership [35]. Using a threshold at 0, the categorial variable *y* had a value of +1 for class “Pasta 100% ITA wheat” and a value of −1 for “Pasta MIX wheat”. Samples were assigned to the class with the highest value of the *y* variable.

#### 2.2.4. Support Vector Machine Classification (SVMc)

The SVMc is a classification method based on statistical learning that uses kernel functions to map from the original space to the feature space. It attempts to find the optimal separation between classes of the training set by fitting them a unique hyperplane [35,38]. The final decision function of SVMc is determined by a small number of support vectors that are the points lying on the margins of the hyperplane. Proper selection of kernel functions is essential to SVMc and affects the performance of the model. In the present paper, among different available functions, the linear kernel type was used to determine the hyperplane separating the two classes with a *nu*-value set at 0.5 (and a threshold set at 0.25), where *nu* serves as the upper bound of the fraction of errors and is the lower bound of the fraction of support vectors.

#### 2.2.5. Evaluation of Classification Performance

The performance of the classification models in terms of sensitivity, accuracy, and specificity was calculated for both the training and the test sets according to the confusion matrices for binary classification [35,39]. By assuming that the class “Pasta 100% ITA wheat” is that of interest, samples are defined as true positive (TP) if they are correctly found as belonging to this class, or false negative (FN) if they are classified as not belonging to it. By analogy, samples of the “Pasta MIX wheat” are defined as true negative (TN) if they are correctly found as belonging to this class, or false positive (FP) if they are classified as not belonging to it.

Sensitivity, calculated for both training and test sets, is defined as the fraction of samples, belonging to the class of interest, which are correctly classified by the model and is a measure of the confidence level of the class space:

*Sensitivity* = [TP/(TP + FN)] × 100

Specificity is defined as the fraction of samples not belonging to the class of interest that are correctly rejected by the model:

*Specificity* = [TN/(TN + FP)] × 100

Accuracy is defined as the fraction of correctly classified samples with respect to the entire set:

*Accuracy* = [(TP + TN)/(TP + TN + FP + FN)] × 100

## 3. Results and Discussion

### 3.1. Spectral Information

**Figure 1:**
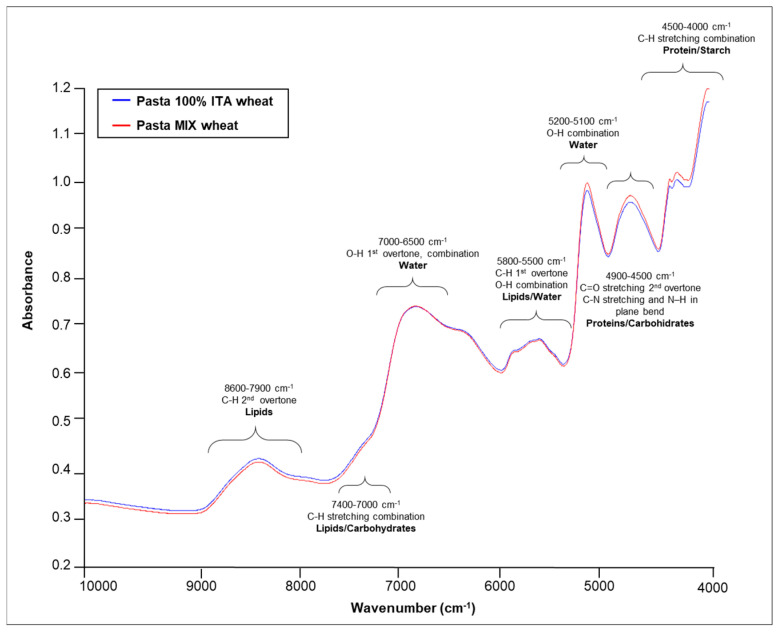
Overlay of average FT-NIR raw spectra of pasta classes, i.e., pasta made with 100% Italian wheat (blue trace) and pasta made with a mixture of wheat cultivated abroad (red trace), with fundamental spectral bands.

The unprocessed average FT-NIR spectra profiles of pasta samples, manufactured with wheat grown exclusively in Italy and of those manufactured with a mixture of wheat cultivated abroad, are presented in Figure 1. The assignment of pasta signals was done through comparison with the list of the basic characterizing wavelengths in the NIR region of different functional groups related to agricultural products described by Shenk et al. [40], Stuart et al. [34], and Manley et al. [41]. By comparing the average FT-NIR raw spectra of the two classes, no visible differences in the shape of the spectra were observed with main absorbance features around 8300 cm^−1^, 6800 cm^−1^, 5300 cm^−1^, and 4000 cm^−1^ (Figure 1).

Specifically, the band between 8600 and 7900 cm^−1^ was attributed to the second overtone of C–H stretching and was ascribed to lipids, while the broad absorption bands at 6800 cm^−1^ and at 5200 cm^−1^ are combination bands related to the hydrogen of water as well as of other hydrogen-containing molecules. The latter band is also associated with the O–H stretching first overtone of starch and to the third overtone of the carbonyl group of proteins. Furthermore, the regions between 7400 and 7000 cm^−1^ and 4350 and 4030 cm^−1^ could be attributed to a combination of stretching and deformation of the C–H group, typically from fatty acids and carbohydrates. The band at 6300 cm^−1^ arose from the first overtone of the O–H stretching of starch, while the band between 5800 and 5500 cm^−1^ was attributed to the first overtone of C–H stretching of lipids and to the O–H combination of water. Finally, the band between 4900 and 4500 cm^−1^ arose from the combination of C = O stretch second overtone and C–N stretching and N–H in-plane bend of both proteins and carbohydrates [34,40,41] (Figure 1). These outcomes confirmed those reported by other authors applying NIR spectroscopy to the traceability of wheat and pasta [24,27,28,30,31,32,33].

### 3.2. PCA

**Figure 2:**
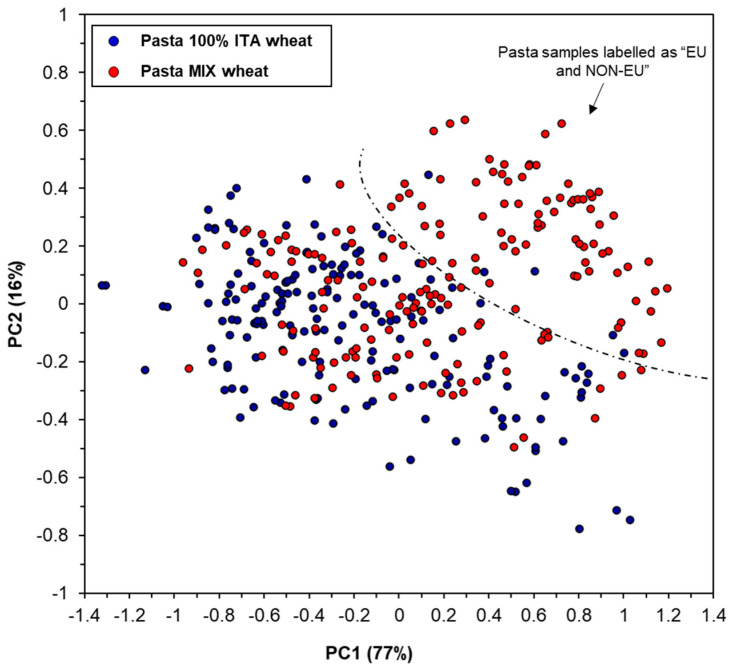
Principal components (PCA) score plot for pasta samples (entire set) analyzed in the spectral range between 10,000–4000 cm^−1^ and using maximum normalization, standard normal variate (SNV), and mean centering pre-processing of spectral data.

Unsupervised PCA by using a total number of 20 PCs was employed on pre-processed spectra for visualizing data trends in a dimensional scatter plot. By plotting the PCA score plot of PC1 vs. PC2 (explaining 77% and 16% of the total variance, respectively), a low level of clusterization of the pasta samples in relation to their wheat origin was found. Specifically, a group of pasta samples manufactured with a mixture of foreign wheat were separated by pasta samples manufactured exclusively with Italian wheat, while the others completely overlapped (Figure 2). This could be explained by the high percentage of Italian wheat used in pasta samples belonging to the class “Pasta MIX wheat”. It should be highlighted that, within this class, 102 samples were labelled as “EU and NON-EU”, and 83 were labelled as “Italy and other EU or NON-EU countries”. This means that the latter pasta samples could contain 50–99% of Italian wheat, thus explaining the observed overlapping between the two classes of samples.

To explore the contribution of original variables to the PCs, we focused on the loading plot, which might reveal regions of high importance for the model (variations related to the geographical origin of wheat). Variables lying within the upper and lower bounds (correlation values between +0.7 and +1 and between −0.7 and −1) were the most important ones modelled by PC1. Specifically, it was observed that only the absorbance range between 7500 and 4000 cm^−1^ was dominant in PC1, showing correlation loading values ≥+0.7. This region was related to lipid, carbohydrates, water, and protein absorption, thus suggesting that it contained most of the information responsible for the discrimination of pasta samples. The Mann–Whitney U Test performed on the content of proteins, carbohydrates, lipids, fiber, and salt indicated that the level of lipids in pasta manufactured with a mixtures of durum wheat (1.5 ± 0.21%) was significantly (*p* < 0.05) different compared to that of pasta manufactured with durum wheat grown exclusively in Italy (1.4 ± 0.32%), thus suggesting the contribute of these compounds to the variability between the two classes. This variability was probably related to the different growing zones, latitudes, and moisture conditions of the wheat used for manufacturing the samples analyzed in the present study, as reported elsewhere [8,9,42]. These results also confirmed those reported by Firmani et al. [32,33] that investigated the variable importance in projection indices to examine which variables were mainly involved in the discrimination between Gragnano and non-Gragnano pasta samples and between durum semolina varieties harvested in different Italian macro-areas. They found that the most relevant spectral zones were around 4000, 5000, and 7000 cm^−1^.

Then, a new PCA was run by using a reduced spectral range, i.e., from 7500 to 4000 cm^−1^. By plotting the PCA scores of PC1 vs. PC2 (explaining 63% and 29% of the total variance, respectively), a slightly better clustering of the pasta samples based on wheat origin was observed, even though some overlapping between the two classes was still observed.

### 3.3. Supervised Classification Models

The supervised classification models used for the authentication of Italian pasta samples manufactured exclusively with durum wheat grown in Italy were PC-LDA, PLS-DA and SVMc. The performance results in terms of sensitivity, specificity, and accuracy were compared to select the best classification approach.

According to the above-mentioned results, a reduced spectral range, i.e., from 7500 to 4000 cm^−1^, was used for developing and validating the three supervised classification models; the results are reported in Table 2. By keeping in mind that the class “Pasta 100% ITA wheat” was that of interest, sensitivity, specificity, and accuracy were calculated accordingly, as reported in Section 2.2.5. Sensitivity rates were between 93 and 98% for the training set and between 88 and 95% for the test set, while the specificity rates of the three models were between 77 and 95% and 82 and 94%, respectively. Finally, the accuracy rates were between 86 and 96% for the training set and between 85 and 94% for the test set (Table 2).

**Figure 3:**
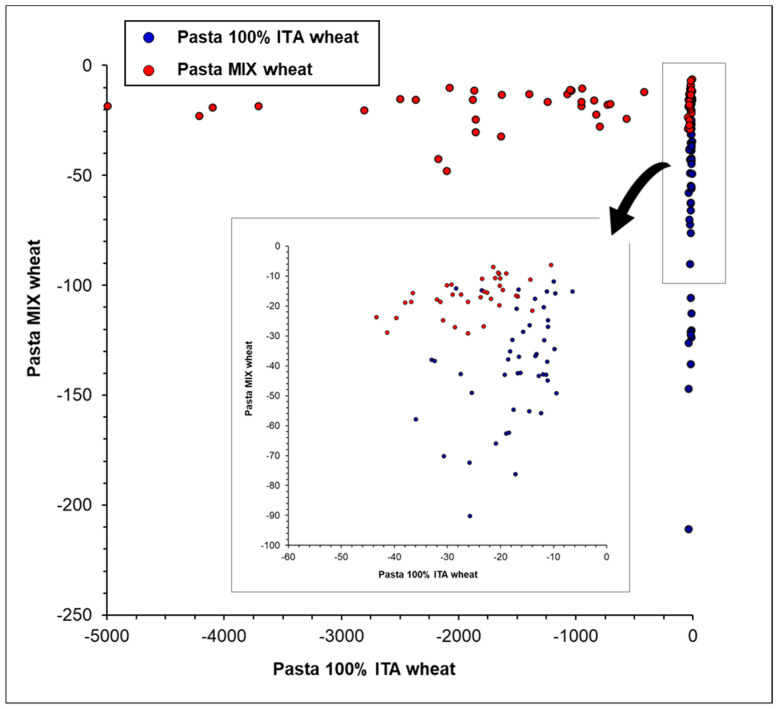
PC-LDA score plot for pasta samples (test set) analyzed by FT-NIR in the spectral region between 7500 and 4000 cm^−1^ using maximum normalization, SNV, and mean centering pre-treatment of spectral data. The insert shows an expanded region of the score plot.

**Figure 4:**
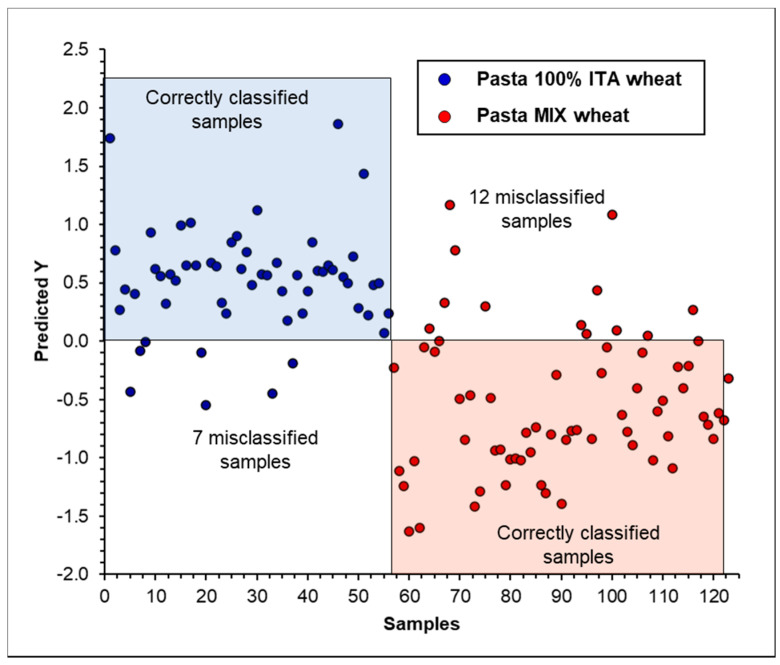
PLS-DA score plot for pasta samples (test set) analyzed by FT-NIR in the spectral region between 7500 and 4000 cm^−1^ using maximum normalization, SNV, and mean centering pre-treatment of spectral data.

By comparison, it was observed that PC-LDA was the best-performing approach, yielding a sensitivity of 98% for the training set and of 95% for the test set and an accuracy of 96% for the training set and of 94% for the test set. The goodness of the model was also confirmed by the specificity of 94% for the test set, indicating the high ability of the model to reject the objects of the other class. The good ability of the model to discriminate the two classes, i.e., “Pasta 100% ITA wheat” and “Pasta MIX wheat”, was also confirmed by the PC-LDA score plot of the test set shown in Figure 3. The observed partial scattering of the class “Pasta MIX wheat” was probably due to the variable percentage of Italian wheat used in these samples, as previously observed for PCA. On the other hand, the worse model was that obtained using the PLS-DA approach, as clearly visible in the score plot showing the numerous misclassified samples in the two classes (Figure 4).

To be sure that the use of the selected range did not affect the final results, the three supervised classification models were also applied on the entire spectral range (i.e., 10,000–4000 cm^−1^). Comparable results in terms of sensitivity and accuracy rates between the three classification models were obtained for both training and validation sets. Specifically, sensitivity rates were between 92 and 97% for the training set and between 87 and 90% for the test set, while accuracy rates were between 92 and 98% and between 86 and 89%, respectively (data not shown). These accuracy results lower than 90% obtained for the test set indicated that, although the entire spectral range could increase the amount of information, worse results were obtained.

The results obtained in the present study are in agreement with those recently reported by Firmani and co-workers [32] on the use of NIR spectroscopy in combination with PLS-DA and soft independent modelling of class analogies (SIMCA) for the authentication of protected geographical indication (PGI) Gragnano pasta, a typical durum pasta produced in a specific area in the South of Italy. Moreover, Biancolillo et al. [29] applied NIR spectroscopy for the determination of turmeric adulteration in egg pasta by using the PLS regression model. Recently, FT-NIR spectroscopy combined with the PC-LDA classification model was successfully applied for tracing the geographical origin of durum wheat samples from different areas of Italy (North, Center, and South) and to discriminate Italian durum wheat samples from those cultivated abroad [23]. Accuracy values up to 100% confirmed the robustness of this classification model. Furthermore, the FT-NIR spectral range between 7700 and 4500 cm^−1^ provided the best performance results, thus suggesting that starch, lipids, and proteins that absorb in this region may be the factors responsible for the geographical origin discrimination of wheat [23]. In another work, NIR spectroscopy was applied for the classification of different cultivars of Italian durum wheat semolina. The fusion of NIR data with alveographic parameters correctly classified 100% of samples based on their geographical origin [33]. NIR spectroscopy, in combination with PC-LDA and PLS-DA, was also successfully applied for the geographical origin discrimination of wheat flour [24,27,28]. Furthermore, very recently, an EU validation procedure for screening methods was successfully applied to a multivariate FT-NIR spectroscopic method for durum wheat pasta authentication. The results of this proof-of-concept strategy demonstrated that FT-NIR is an attractive and suitable tool for the detection of durum wheat pasta adulteration [31].

## 4. Conclusions

This paper describes, for the first time, the application of FT-NIR spectroscopy, combined with chemometrics, for the authentication of Italian pasta made exclusively with durum wheat cultivated in Italy. To achieve this goal, more than 350 pasta samples marketed in Italy were analyzed by FT-NIR coupled with supervised classification models, i.e., PC-LDA, SVMc, and PLS-DA, and the performance results were compared. The analysis of correlation loadings indicated that the FT-NIR region in the range between 7500 and 4000 cm^−1^ contained most of the information useful for the discrimination of pasta samples based on the geographical origin of wheat, which was also indicated by the lipid content that was significantly different among the two classes. PC-LDA provided the highest accuracy values, while PLS-DA showed the lowest ones. Furthermore, the PC-LDA model demonstrated to be particularly suitable to reject samples not belonging to the class “Pasta 100% ITA wheat”, thus resulting as the best discriminant approach.

The outputs of this work showed that FT-NIR spectroscopy, coupled with a suitable multivariate classification model, i.e., PC-LDA, is a reliable and robust tool that can be used for authenticating durum wheat pasta samples based on the geographical origin of wheat, with a low probability of discrimination error. These results will certainly have a great impact for both Italian pasta producers founding their business on local authenticated products and consumer associations highly interested in preserving and promoting the typicity of Italian pasta and other classes of products for their high added value.

## Figures and Tables

**Table 1:** Kennard–Stone splitting of the data for training and prediction. Pasta 100% ITA wheat, containing only durum wheat cultivated in Italy, Pasta MIX wheat, containing a mixture of wheat cultivated in Italy and/or abroad.

Classes	Pasta 100% ITA Wheat	Pasta MIX Wheat
Total samples	176	185
Training set	120	118
Test set	56	67

**Table 2:** Performance parameters (sensitivity, specificity, and accuracy) of the principal component-linear discriminant analysis (PC-LDA), support vector machine classification (SVMc), and partial least-squares discriminant analysis (PLS-DA) for both the training and the test sets of pasta samples obtained in the spectral range between 7500 and 4000 cm^−1^.

Sample Set	Classification Models	Sensitivity	Specificity	Accuracy
Training set	PC-LDA	98%	95%	96%
	SVMc	93%	85%	89%
	PLS-DA	94%	77%	86%
Test set	PC-LDA	95%	94%	94%
	SVMc	96%	82%	89%
	PLS-DA	88%	82%	85%
